# MTM: a multi-task learning framework to predict individualized tissue gene expression profiles

**DOI:** 10.1093/bioinformatics/btad363

**Published:** 2023-06-05

**Authors:** Guangyi He, Maiyue Chen, Yingnan Bian, Ence Yang

**Affiliations:** Department of Medical Bioinformatics, School of Basic Medical Sciences, Peking University, Beijing 100191, China; School of Artificial Intelligence, Peking University, Beijing 100191, China; Enlight Medical Technologies (Shanghai) Co., Ltd, Shanghai 201318, China; Department of Medical Bioinformatics, School of Basic Medical Sciences, Peking University, Beijing 100191, China; Chinese Institute for Brain Research, Beijing 102206, China

## Abstract

**Motivation:**

Transcriptional profiles of diverse tissues provide significant insights in both fundamental and translational researches, while transcriptome information is not always available for tissues that require invasive biopsies. Alternatively, predicting tissue expression profiles from more accessible “surrogate” samples, especially blood transcriptome, has become a promising strategy when invasive procedures are not practical. However, existing approaches ignore tissue-shared intrinsic relevance, inevitably limiting predictive performance.

**Results:**

We propose a unified deep learning-based multi-task learning framework, multi-tissue transcriptome mapping (MTM), enabling the prediction of individualized expression profiles from any available tissue of an individual. By jointly leveraging individualized cross-tissue information from reference samples through multi-task learning, MTM achieves superior sample-level and gene-level performance on unseen individuals. With the high prediction accuracy and the ability to preserve individualized biological variations, MTM could facilitate both fundamental and clinical biomedical research.

**Availability and implementation:**

MTM’s code and documentation are available upon publication on GitHub (https://github.com/yangence/MTM).

## 1 Introduction

Gene expression profiles in diverse tissues act as molecular mediators between genotypes and phenotypes and provide a snapshot of the systemic physiological and pathological status of an individual (Collins and Varmus 2015). Deciphering tissue-derived gene expression not only provides insights into the fundamental molecular mechanisms of biological processes (Ji *et al.* 2004, Lage *et al.* 2008, Kutsenko *et al.* 2014), but also aids in clinical diagnosis, subtyping, and management (Golub *et al.* 1999, Irgon *et al.* 2010, Hoadley *et al.* 2014). Tissue transcriptome-based biological investigations and clinical evaluations highly depend on tissue biopsy samples. However, biopsy procedures are often costly, invasive, and even infeasible in some cases. More importantly, invasive biopsy is also associated with increased infections and complications (Lundström *et al.* 2014, Di Meo *et al.* 2017), and thus presents more risks especially for pediatric and geriatric populations (Hassan *et al.* 2012, Greenhalgh *et al.* 2014, García-Albéniz *et al.* 2017, Pocienė *et al.* 2022). Thus, mapping biological information from more accessible “surrogate” samples to tissue transcriptomic profiles has become an emerging solution attracting extensive research interests (Gamazon *et al.* 2015, Halloran *et al.* 2015, Gusev *et al.* 2016, Wang *et al.* 2016, Barbeira *et al.* 2019, 2018, Hu *et al.* 2019, Wainberg *et al.* 2019, Xu *et al.* 2020, Zhou *et al.* 2020, Basu *et al.* 2021), providing wide prospects for characterization, screening, long-term monitoring, and management of diseases (San Lucas *et al.* 2016, Di Meo *et al.* 2017, Watts 2018).

Various methods have been developed to predict tissue-specific expression for specific individuals. Early attempts utilized genotypes with the rationale that individual variance in genetically regulated expression (GReX) components of tissue-specific gene expression could be explained by genotypes to some extent, i.e. expression quantitative trait loci (eQTL) (Gamazon *et al.* 2015, Gusev *et al.* 2016, Barbeira *et al.* 2018, Wainberg *et al.* 2019). A representative work demonstrating this strategy is the PrediXcan, which takes advantage of eQTL single-nucleotide polymorphisms (eSNPs) to construct gene-based tests to impute GReX (Gamazon *et al.* 2015). However, these eQTL-based methods are limited to small proportions of genes with significant tissue-specific eSNPs and neglect non-GReX components in expression (Gamazon *et al.* 2015). In contrast, since blood is involved in the circulation and bidirectional exchange of substances with tissues and organs, it is considered to reflect the real-time functional status of tissues underlying gene expression, including non-GReX components from feedback of traits, and environmental and other factors (Liew *et al.* 2006, Mohr and Liew 2007). Furthermore, recent studies have supported the advantages of predicting tissue expression profiles based on blood expression (Halloran *et al.* 2015, Wang *et al.* 2016, Xu *et al.* 2020). Nevertheless, the methods based on genotype and transcriptome usually have two common limitations: first, they are typically based on linear models or traditional machine learning methods, inevitably limiting their capability to capture complex nonlinear expression relationships in biological organisms (Von Bertalanffy 1957, Hasty *et al.* 2001); second, they build prediction models independently for a single gene in each target tissue, failing to utilize the intrinsic cross-tissue biological inherence underlying transcriptomes of multiple tissues from the same individual, which could be rescued by borrowing information from similar tissues (Barbeira *et al.* 2019, Hu *et al.* 2019, Zhou *et al.* 2020).

Deep learning has shown its powerful capability for handling high-dimensional, complex, and nonlinear biological data (Lopez *et al.* 2018, Maj *et al.* 2019, Marouf *et al.* 2020, Park *et al.* 2020, Azevedo *et al.* 2021, Elmarakeby *et al.* 2021, Viñas *et al.* 2021, 2022). Multi-task learning, which imposes regularity or shared representation by learning tasks simultaneously, is ideal for utilizing the intrinsic shared features of different domains (e.g. different tissues from the same individual) (Caruana 1997, Choi *et al.* 2017, Ruder 2017, Yang *et al.* 2019).

Here, we developed multi-tissue transcriptome mapping (MTM), a deep learning-based multi-task learning framework to predict individualized tissue gene expression profiles using any available tissue from a specific person. By jointly leveraging individualized cross-tissue information from multi-tissue reference samples through multi-task learning, MTM achieves superior sample-level and gene-level accuracy (1.63 and 2.29 times higher, respectively), and larger proportions of predictable genes (1.87 times more) than existing methods on unseen individuals. Meanwhile, fine-grained, individualized physiological, and pathological variations are well preserved, with the mean correlation of age-related expression changes up to 0.81. Besides, predicting arbitrary target tissue from arbitrary input tissue in a unified model is supported, which introduces more flexibility and bypasses the need to establish independent models for all prediction directions (which requires quadratic space complexity). With its outstanding predictive power and the ability to capture individualized physiological and pathological variations, MTM demonstrates its potential to accelerate transcriptome-based research and clinical applications.

## 2 Methods

### 2.1 Data preparation for MTM

The gene expression data from human tissues were downloaded from the v8 release of the Genotype-Tissue Expression (GTEx) portal (https://www.gtexportal.org/) (GTEx Consortium 2017). The metadata, including the subject-level and sample-level information, were obtained through the database of Genotypes and Phenotypes (dbGaP) (https://www.ncbi.nlm.nih.gov/gap/). Gene expression levels (transcripts per million, TPM) of 19 291 protein-coding genes across 49 tissues (sample sizes greater than 50) from 944 individuals (individuals with at least two samples, resulting in 17 329 samples in total) were used for model development. The tissue expression profiles were split into the training set and the validation set based on corresponding individual labels (individuals were randomly split into 80% for training and 20% for validation). *Z*-score standardization was conducted within each tissue for the training data, and then the recorded scaling factors were used for the validation data to prevent information leakage.

### 2.2 Description of MTM

**Figure 1.**
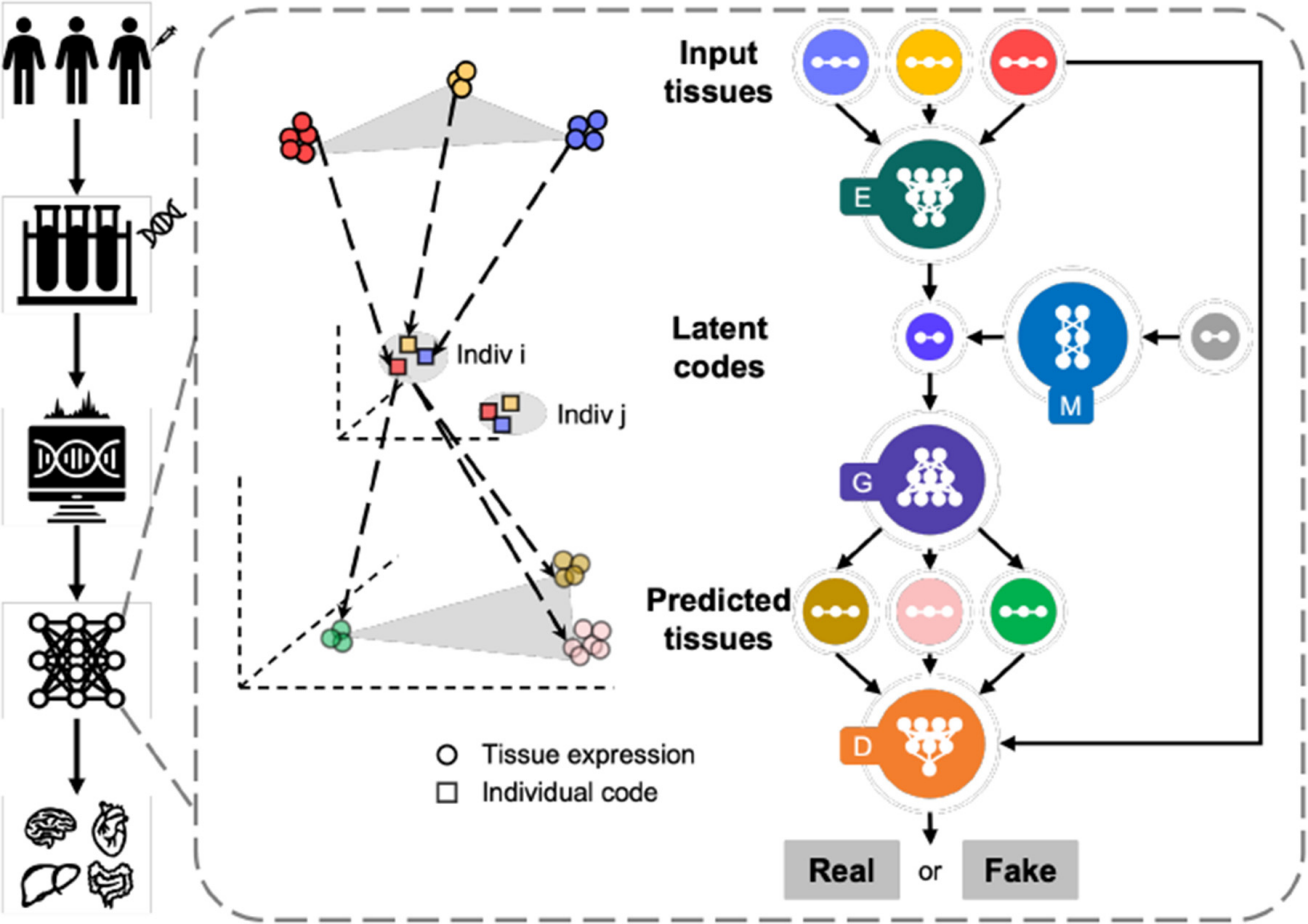
Overview of MTM. MTM is based on a multi-task learning architecture and consists of four artificial neural networks, a unified encoder (*E*), a unified generator (*G*), a unified discriminator (*D*), and a mapping network (*M*). The gene expression profiles of different input tissues are embedded into a shared latent space by *E* to form individualized codes, which are then mapped to multiple tissue-specific expression spaces by *G* to realize personalized prediction of the expression profiles of different target tissues.

**Framework overview.** To achieve individualized gene expression prediction, we proposed a deep learning framework, MTM, a unified multi-task learning approach capable of predicting tissue-specific gene expression profiles using any available tissues from an individual (Fig. 1). The expression levels of input tissue samples across different tissues were processed by a unified encoder network equipped with the tissue conditioning module (TCM), to encourage personalized representations in a shared latent space. Then, the latent codes were mapped to multiple tissue-specific expression spaces through a prediction task conditioning generator to achieve personalized prediction of tissue expression profiles. Two auxiliary networks, a mapping network and a discriminator network, were employed to smooth the latent space and to refine the prediction results, respectively. With the multi-task learning architecture, MTM is not limited to predicting the expressions of a single target tissue based on a single input tissue (single mapping direction), but rather supports arbitrary input tissues and arbitrary target tissues (arbitrary mapping directions) in a unified model, which introduces more flexibility along with potentially improved performance.

**Key elements.** The MTM architecture consists of an encoder, a generator, a discriminator, and a mapping network (Fig. 1). The encoder (*E*) embeds expression profiles into a shared latent space, resulting in personalized representations, i.e. latent codes. The generator (*G*) is symmetrical to the structure of the encoder, through which the latent codes were mapped into tissue-specific expression spaces in the form of individualized tissue expression profiles. The discriminator (*D*) is employed to identify the mismatches of the predicted distributions and the actual distributions to send feedback signals for the generator to improve the prediction in an adversarial manner (Goodfellow *et al.* 2020). The auxiliary mapping network (*M*) assists *E* in learning a smooth space (Karras *et al.* 2018). All *E*, *G*, and *D* networks are multi-task networks equipped with TCMs, which were designed to route and transform the data through tissue-specific or shared connections. The TCM consists of three parts: a shared fully connected layer and a learnable instance-level affine transformation conditioned on tissue labels followed by a leaky rectified linear unit (Leaky ReLU) to introduce nonlinearity. The detailed structures of the MTM are described in Supplementary Figs S1–S5.

**Objectives.** The generator (G) translates latent codes from a shared latent space (c∈W) to predict the expression profiles conditioned on the target tissue $t,G(c|t)\to x_{t}$. The latent codes could either be reference-guided by E(E(x∣s)→*c*) or transformed from random Gaussian noise z∼N(0,1) by $M(M(z)\to \tilde{c})$. To enforce *G* to utilize the latent code $\tilde{c}$ when predicting the expression profile $G(\tilde{c}|t)$, we employed the L1 reconstruction loss:


(1) $$L_{R}=E\left[\|E(G(\tilde{c}|t)|t)-\tilde{c}{\|}_{1}\right].$$

To minimize the divergence between the distributions of the actual expression profiles and of the predicted ones (G(⋅)), a training strategy from a generative adversarial net (G|AN) is used, where *D* tries to distinguish the generated samples from real samples, while *G* tries to fool *D*. Instead of the original GAN, which minimizes the Jensen–Shannon divergence (Goodfellow *et al.* 2020), we used an adversarial objective function in the form of the hinge loss for robust training (Lim and Ye 2017, Gui *et al.* 2023):


(2) $$\begin{array}{l} \begin{array}{l} L_{adv}(D)=E_{x∼p_{\text{data }}(x)}[\max(0,1-D(x|t))] \end{array}\\ +E_{z∼p_{z}(z)}[\max(0,1+D(G(z|t)))] \end{array}$$

(3) $$L_{adv}(G)=-E_{z∼p_{z}(z)}[D(G(z|t))]$$

To achieve individualized tissue expression prediction, given the expression profile of the source tissue $x_{s}$ from a specific individual, we use the L1 loss to penalize the difference between the predicted expression profiles $G\left(E\left(x_{s}|s\right)|t\right)$ and the actual expression profile $x_{t}$ of the target tissue *t*:


(4) $$L_{I}=E\left[{\left\|G\left(E\left(x_{s}|s\right)|t\right)-x_{t}\right\|}_{1}\right].$$

To ensure that the predicted expression profile properly preserves the individual invariant characteristics, a cycle consistency loss is employed:


(5) $$L_{C}=E\left[{\left\|G\left(E\left(G\left(E\left(x_{s}|s\right)|t\right)|t\right)|s\right)-x_{s}\right\|}_{1}\right].$$

The final objective function is as follows:


(6) $$L=L_{\mathrm{adv}}+\lambda_{R}L_{R}+\lambda_{I}L_{I}+\lambda_{C}L_{C}.$$

**Training process.** In the training process, data are organized in the form of tissue pairs within the same individuals, where one is used as input and the other as prediction target. In this way, MTM is trained to learn the mapping between any two types of tissues, hence improving the prediction performance of each direction. Each fold of the 5-fold cross-validation (CV) process requires an average of 7.97 h, 20.8 GB RAM, and 4.8 GB GPU RAM during training. All deep learning-based models were trained using the PyTorch framework (version 1.10.2) (Paszke *et al.* 2019) on Tesla V100S graphics processing units (NVIDIA).

**Training hyperparameters.** The data were split into mini-batches of 256 samples for training. The Adam optimizer (Kingma and Ba 2014) was employed to optimize the network parameters using the hyperparameters recommended by implementations from Warde-Farley and Bengio (2017), Miyato *et al.* (2018), Lewkowycz *et al.* (2020), and Beugnot *et al.* (2022): learning rate =0.0005, β1=0.5, and β2=0.9. The number of maximum training epochs was set to 200 to avoid overfitting.

### 2.3 Description of compared single-tissue methods for ablative experiments

B-GEx (Method S1) and TEEBoT (Method S2) use linear regression models to predict tissue gene expression from blood sample gene expression features (Xu *et al.* 2020, Basu *et al.* 2021). Each method creates a linear regression model for each gene in each target tissue. S1 employs a Bayesian ridge regression model with the most relevant subset of features identified through feature selection for each target gene, while S2 builds a least absolute shrinkage and selection operator (LASSO) model with principal components of the blood expression data as features. Method S3 consisted of a set of nonlinear neural networks for each tissue based on simple multilayer perceptrons. For each tissue, a fully connected neural network with Leaky ReLU activations was built to predict the expression profiles of the target tissue from the expression of a specific source (blood). The number of maximum training epochs was set to 1000. Other training hyperparameters were kept the same as those of MTM.

### 2.4 Performance evaluation

In the evaluation process, only gene expression profiles of blood samples are used as input and other tissues as output. The prediction quality was indicated by the Pearson correlation coefficient (Pearson’s ρ) between the predicted and observed expression data. The sample-wise accuracies across all genes were calculated on *Z*-score standardized data to highlight the individual variance in each tissue. The gene-wise accuracies refer to Pearson’s ρ of each gene across individuals for each tissue. Predictable genes (pGenes) were defined as genes with gene-wise ρ > 0.3 between predicted and observed expression in each tissue. Accuracy indicators in this work were reported in a 5-fold CV manner, where predicted data were concatenated for the following evaluation. For fair comparison, the performance of all models was evaluated in a CV manner using the same inputs as were used with MTM (blood expression data from the same training and validation samples).

### 2.5 Exploration of the characteristics of predictable genes

**Tissue similarity.** Similarities between tissues were measured with Pearson’s ρ across genes between the mean expression levels averaged over individuals of each tissue (${\;\text{log}}_{2}$-scaled). When comparing certain pairwise statistics (x1) of tissues with the tissue similarities (x2), element-wise values were flattened to two arrays (X1 and X2), and then Spearman’s correlation coefficient (Spearman’s ρ) between the two was calculated.

**Expression levels.** In each tissue, the Wilcoxon rank sum test was used to compare the mean expression levels of the top 25 percentiles of predictable genes (highly predictable genes) with those of the control groups, with the control groups defined as the bottom 25 percentiles of predictable genes (unpredictable genes).

**Conservation.** The conservation distributions of genes in terms of PhastCons scores were extracted with DeepTools (Ramírez *et al.* 2016) from the 100-way vertebrate species alignment downloaded from the UCSC (University of California Santa Cruz) genome browser (Siepel *et al.* 2005, Haeussler *et al.* 2019). The conservation levels of the highly predictable genes were compared with those of the unpredictable genes in each tissue (Wilcoxon rank sum test).

**Connectivity.** The degree of connectivity distribution for each gene in the protein interaction network (PIN) was downloaded and extracted from HIPPIE v2.3 (Alanis-Lobato *et al.* 2017). Then, the average degrees of the two groups of genes (highly predictable genes and unpredictable genes) were compared. The average degrees of the highly predictable genes were compared with those of the unpredictable genes in each tissue (Wilcoxon rank sum test).

### 2.6 Exploration of the characteristics of the intermediate features of MTM

Personalized representations from the latent space of the MTM encoder were extracted across different individuals and tissues and then used to calculate pairwise similarities (Pearson’s ρ). Intraindividual codes refer to latent codes of different tissues from the same individual, while interindividual codes refer to latent codes from different individuals. Decoding paths of the data flow from latent codes to tissue expression spaces refers to activations of different layers through the MTM generator. The decoding paths of each layer from an individual’s code from blood to different tissues were used to calculate pairwise similarities (Pearson’s ρ). Spearman’s ρ between the pairwise similarities of the decoding paths and the pairwise tissue similarities was calculated to determine the extent to which the decoding rules reflected biological patterns.

### 2.7 Phenotype-related analysis

For traits including age, gender, and BMI, Pearson’s ρ was used to measure the associations of each gene with traits in each tissue. Then, the Pearson’s ρ between the predicted and actual (GTEx) associations with traits was calculated to determine the extent to which the predicted tissue expression preserved the trait-related expression changes.

For disease-associated dysregulations, the sign consistency and Pearson’s ρ between ${\;\text{log}\;}_{2}$ fold changes across genes in predicted and actual data were used to measure the overall concordance of the prediction. Differential expression analysis (DE analysis) was conducted using the nonparametric Wilcoxon rank sum test. When comparison of differentially expressed genes (DEGs, under FDR < 0.05) between predicted and actual data was applicable, hypergeometric tests were conducted to examine the overlap significance, and the F1 scores, which convey the balance between precision and recall, were used to measure the performance of identifying DEGs.

For disease status prediction, we built LASSO models with scikit-learn (version 0.24.2) (Pedregosa *et al.* 2011) for each disease–tissue pair in the predicted expression, the actual expression, and the input blood expression. The values of the area under the ROC curve (auROC) were used to indicate performance in a 5-fold CV manner with 50 independent repetitions.

### 2.8 Exploration of MTM applications on external datasets

The raw sequencing data (fastq files) of blood expression profiles from GSE184050 (Chen *et al.* 2022) were downloaded and the gene expression values of the 19 291 protein-coding genes were quantified according to the pipeline provided by the GTEx Consortium (2017). To reduce batch effects, we simply aligned the mean and standard deviation of each gene in each tissue to those of the GTEx data. For external dataset, the MTM was trained in a hold-out manner, with 80% of the data used for training and the remaining 20% for testing.

The standardized blood expression profiles of GSE184050 (type 2 diabetes, T2D) were input into MTM to predict the tissue expression profiles of corresponding individuals. Then, Pearson’s ρ between ${\;\text{log}}_{2}$ fold changes across genes in predicted data and reference data (case and control subjects for T2D in GTEx) was used to measure the overall concordance of the predicted disease-related dysregulations.

## 3 Results

### 3.1 MTM outperforms the baseline methods

**Figure 2.**
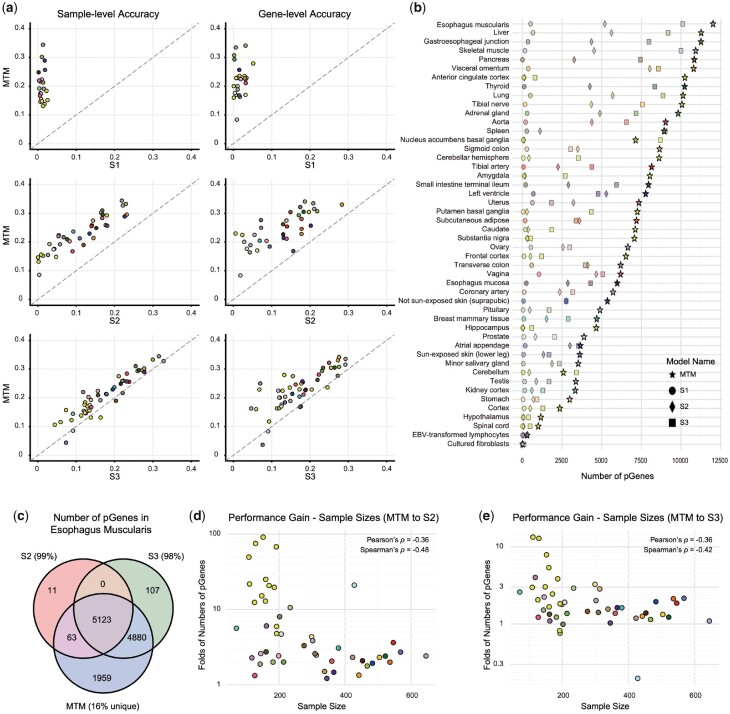
Comparison of the performance for predicting tissue-specific gene expression. (a) The sample-wise and gene-wise accuracy (Pearson’s correlation coefficient, Pearson’s ρ) of MTM against single-tissue approaches, including linear (S1 and S2) and nonlinear (S3) models. The values represent the mean performance of all individuals (sample-level) or of all genes (gene-level) in the target tissue. (b) The number of predictable genes (pGenes, defined as gene-wise Pearson’s ρ > 0.3) of different models in different target tissues. (c) Venn diagram showing the overlap of the pGenes from S2, S3, and MTM in the esophagus muscularis. Approximately 99% and 98% of pGenes from S2 and S3 were included in the MTM-derived pGenes, respectively. (d, e) The relationship between the fold changes in the number of pGenes and the sample sizes of different tissues for MTM to S2 (d) and MTM to S3 (e). Each scatter point color represents a specific tissue as defined by the GTEx Consortium (GTEx Consortium 2017).

Gene expression levels (TPM) of 19 291 protein-coding genes across 49 tissues (sample sizes greater than 50) from 948 individuals (17 329 samples in total) in the GTEx (GTEx Consortium 2017) datasets were used to develop our model. The prediction quality of MTM at the sample level and the gene level was evaluated on the task of predicting individualized gene expression profiles of the other 48 tissues within the development set (GTEx) based on the expression profile of whole blood. Two representative single-tissue approaches, B-GEX (Method S1) (Xu *et al.* 2020) and TEEBoT (Method S2) (Basu *et al.* 2021), were used as baselines in the evaluation for comparison. At the sample level, the sample-wise prediction accuracy was measured by Pearson’s ρ, which was 0.21±0.07 across all genes among the 48 tissues (ranging from 0.04 to 0.34). Compared with 0.00±0.02 for S1 and 0.08±0.09 for S2, MTM showed significantly higher levels of accuracy (Wilcoxon rank sum test, *P*-value < .05) than S1 or S2 did in all 48 tissues. On the gene scale, MTM (0.23±0.07, ranging from 0.04 to 0.34) also demonstrated significantly higher gene-wise accuracy (Wilcoxon rank sum test, *P*-value < .05) than S1 (0.00±0.03) and S2 (0.07±0.11) did in 48 and 47 tissues out of the 48 tissues, respectively (Fig. 2a and Supplementary Table S1).

In terms of predictable genes (pGenes; defined as genes with Pearson’s ρ > 0.3 between predicted and observed expression), MTM on average identified 6597 pGenes across the 48 tissues (ranging from 21 to 12 025), which was substantially higher than the 218 (ranging from 0 to 1048) of S1 and the 2301 (ranging from 1 to 8031) of S2 (Fig. 2b and Supplementary Table S2). For the S1- and S2-derived pGenes, a median of 94.2% (7.7%–100.0%) and 94.5% (0.0%–99.8%) was identified by MTM, respectively. In contrast, a median of 2.2% (0.0%–15.9%) and 32.6% (0.0%–73.1%) of MTM-derived pGenes was captured by S1 and S2, respectively. When comparing the fold changes in the number of pGenes of MTM to S2 (ranging from 1.22- to 91.37-fold, mean = 11.39-fold, median = 2.91-fold), we found that the performance gain was negatively correlated with sample size (Spearman’s ρ=−0.48, $P-\text{value}=5.26\times 10^{-4}$, Fig. 2d), implying that the missing information caused by small sample sizes could be partially recovered from other tissues with the application of MTM.

### 3.2 Both the nonlinearity and multi-task framework contribute to performance

To investigate the independent contribution of the nonlinear and multi-task components, we performed ablative experiments and assessed their influence on the overall performance. We constructed an intermediate model (S3) for comparison, which consisted of a set of nonlinear neural networks for each tissue based on simple multilayer perceptrons.

For the nonlinearity component, we compared the performance of the linear models (S2) with the constructed nonlinear models (S3) for each tissue. As expected, the accuracy of S3 outperformed S2 both at the sample and gene levels (0.17±0.07 and 0.17±0.07, respectively, Supplementary Fig. S6). The sample-wise accuracies and gene-wise accuracies of S3 were significantly (*P*-value < .05) higher than those of S2 in 46 tissues and 43 tissues, respectively. Compared with S2, the average number of pGenes resulting from S3 (on average 4057 pGenes, ranging from 106 to 10 110, Supplementary Table S2) was substantially higher, with fold changes ranging from 0.57- to 106.00-fold (mean = 7.11-fold, median = 1.93-fold).

For the multi-task learning component, MTM was compared with the constructed nonlinear single-tissue models (S3). At the sample level, the accuracy of MTM outperformed S3 in 41 tissues, with the performance in 24 tissues showing statistical significance (*P*-value < .05). In addition, at the gene level, MTM significantly (*P*-value < .05) outperformed S3 in 42 tissues (Fig. 2a and Supplementary Table S1). Furthermore, the average number of pGenes resulting from MTM (6597) was substantially higher than that of S3 (4057) (Fig. 2b and Supplementary Table S2), with fold changes ranging from 0.20- to 13.38-fold (mean = 2.46-fold, median = 1.65-fold). Approximately 92.4% of the S3-derived pGenes were still captured by MTM (Fig. 2c and Supplementary Fig. S7). The above results suggested that apart from advantages introduced by nonlinear neural networks, jointly leveraging the individualized cross-tissue information provides extra prediction improvements perpendicularly.

### 3.3 Intermediate features and outputs of MTM provided insights into the performance basis

To determine how MTM integrated different tissues from the same individual as an entirety, we next explored the characteristics of the individualized representations learned by MTM. Intraindividual latent codes derived from the same individual showed significantly ($P-\text{value}<2.2\times 10^{-16}$) higher similarities than interindividual latent codes did (Supplementary Fig. S8), indicating that individualized properties of different tissues from the same individual were captured well by MTM. Then, we explored the characteristics of the decoding paths from the same individual representation to the tissue expression profiles. We found that the pairwise similarities between the decoding paths of the data flow (i.e. activations of different layers) were highly consistent (Spearman’s *P*-value < .05) with those between tissue expression profiles (Supplementary Fig. S8), suggesting that the biological similarities between tissues were implicitly encoded in the decoding process. Together, the integration of multiple tissues into a single model by our multi-task learning architecture resulted in individualized representations and decoding rules with biological patterns.

Given that MTM is able to identify more pGenes, distinctive features of the pGenes may also aid in the interpretation of the overall prediction strength of MTM from a different perspective. We compared the pairwise similarities of the predictability of genes of different tissues (Supplementary Fig. S9), and found that tissues with similar expression profiles tended to share similar pGenes (Spearman’s ρ = 0.56, $P-\text{value}=1.40\times 10^{-92}$), which was consistent with previous discoveries. The pGenes tended to have significantly (Wilcoxon rank sum test, *P*-value < .05) higher expression levels than the unpredictable genes in all 48 tissues (Supplementary Fig. S10). The pGenes showed significantly (Wilcoxon rank sum test, *P*-value < .05) higher conservation than the unpredictable genes in 9 tissues but significantly lower conservation in 35 out of the 48 tissues (Supplementary Fig. S11), suggesting that there was no obvious preference for conservation in pGenes. Notably, there were significantly higher levels of connectivity (average degrees) in the PIN for pGenes than for unpredictable genes in a large portion of tissues (higher in 47 tissues, with 28 tissues showing statistical significance, while significantly lower in one tissue, Wilcoxon rank sum test) (Supplementary Fig. S8). By comparison, the S2-derived pGenes showed significantly lower levels of connectivity in 11 tissues, while exhibiting significantly higher levels in 26 tissues. The difference between MTM and S2 suggested that MTM might be more capable of capturing the complex interactions in the PIN.

### 3.4 MTM could capture personalized biological variations

As MTM resulted in highly personalized representations of individuals, we assessed the extent to which the predicted tissue expression profiles could reveal trait-associated variations. Correlation analysis of gene expression levels of pGenes was conducted against individuals’ traits (including age, gender, and BMI) in the predicted data (from blood expression) and compared with the actual data (i.e. observed data). The age-specific gene expression associations were well preserved (Pearson’s ρ > 0.3, *P*-value < .05) in the predicted expression data in 46 out of the 48 tissues, with a $\rho_{\text{median}}$ of 0.81. Similarly, the test statistics of gender ($\rho_{\text{median}}$ = 0.76) and BMI ($\rho_{\text{median}}$ = 0.56) of the predicted data showed significant positive (Pearson’s ρ > 0.3, *P*-value < .05) correlations with those of the actual data in all 43 gender-independent tissues and 39 out of the 48 tissues, respectively (Supplementary Figs S12–S14). The above results suggested that the individualized tissue expression profiles predicted by MTM preserved the trait-related expression changes well.

**Figure 3.**
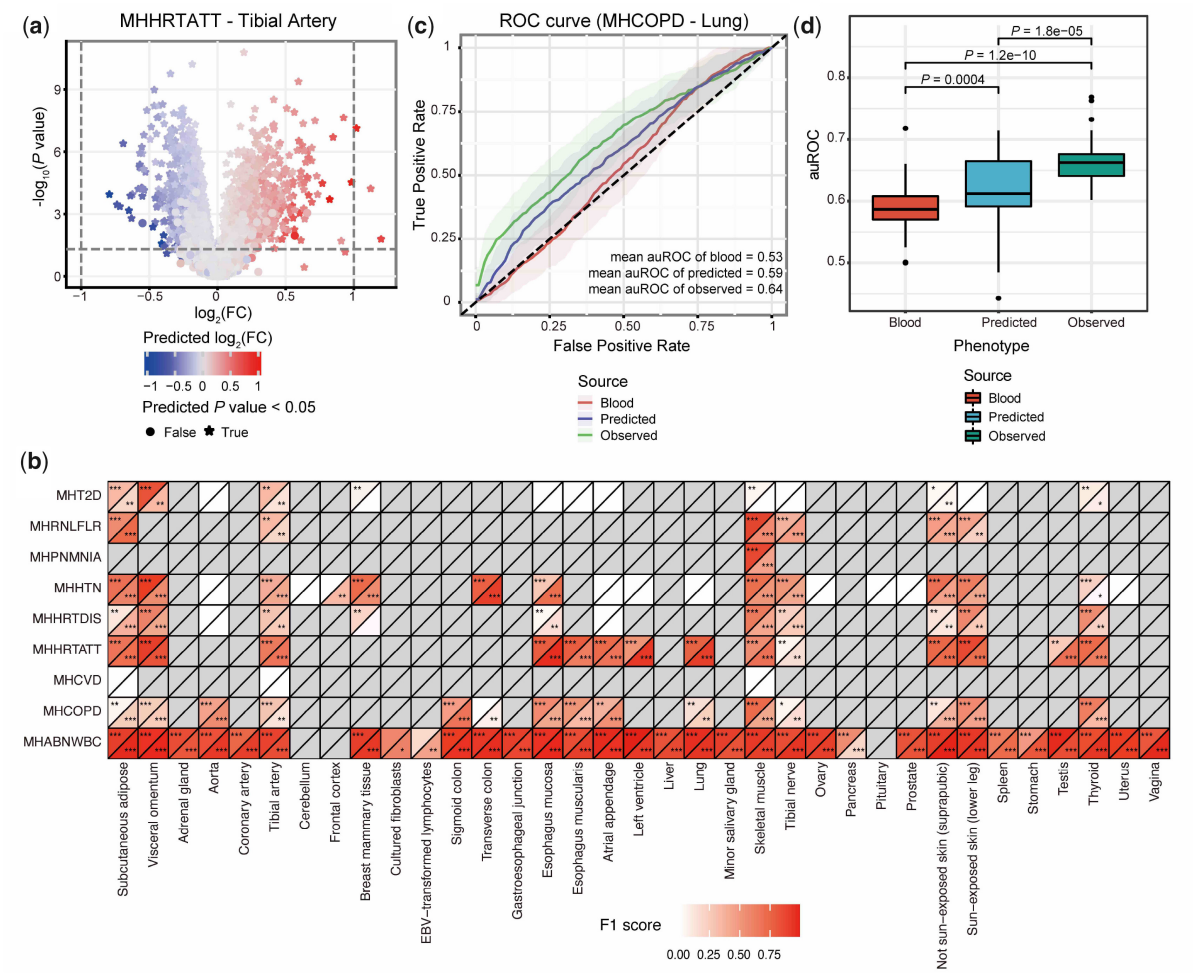
Predicted data preserve personalized biological variations. (a) Comparison of results in DE analysis between the predicted expression and the observed tibial artery expression with heart attack. The position of each point represents the observed results, while the color indicates the predicted ${\;\text{log}}_{2}$ fold change. (b) Performance of predicting DEGs of different diseases in different tissues using predicted data. The number of stars in the grids indicates the overlap significance level of up- or downregulated genes between observed and predicted data (* = significant at .05, ** = significant at $1\times 10^{-10}$ and *** = significant at $1\times 10^{-100}$). The color represents the level of the *F*1 score for predicting DE genes. (c) A specific case of receiver operating characteristic (ROC) curves for predicting chronic obstructive pulmonary disease (COPD) status in the observed lung expression, the predicted lung expression, and the input blood expression. (d) Performance of predicting disease status (auROC) of different cases using observed tissue data, predicted tissue data, and input blood data.

Apart from trait-associated changes, we investigated the consistency between tissue-specific disease-related dysregulations in the predicted data and those of the actual data (GTEx). DE analysis in pGenes was conducted for the combination of each tissue and each disease with at least 50 cases and 50 controls annotated in GTEx, resulting in 117 disease-related tissue–disease pairs across 35 tissues and 9 diseases (with at least 10 DEGs, FDR < 0.05). In these 117 disease-related pairs, 112 pairs exhibited both high sign consistency (sign consistency > 0.7) and high correlation (Pearson’s ρ > 0.3) of ${\;\text{log}}_{2}$ fold changes of pGenes, among which DEGs showed even higher concordance (Supplementary Table S3), suggesting that MTM captured both the direction and the level of the disease-related expression changes. Among 109 pairs with at least 10 observed upregulated DEGs, the predicted DEGs significantly overlapped (hypergeometric test, *P*-value < .05) with the reference DEGs in 93 pairs (*F*1_median_ = 0.65, *F*1 score > 0.7 in 44 pairs). For downregulated DEGs, the predicted DEGs significantly overlapped with reference DEGs in 94 pairs (*F*1_median_ = 0.60, *F*1 score > 0.7 in 41 pairs) among 112 disease-related pairs (with at least 10 observed down-regulated DEGs, Fig. 3a and b).

To assess whether the predicted tissue expression profiles could be employed in indicating disease status, we next compared the prediction performance of the predicted tissue expression, the actual tissue expression, and the input blood expression profiles. Among the 117 disease-related pairs mentioned above, 53 pairs were focused where the actual tissue expression profiles were more informative than those of blood for disease status prediction with auROCs of at least 0.6. Although less predictive (Wilcoxon paired test, $P-\text{value}=1.82\times 10^{-5}$) than the actual tissue expression (${\text{auROC}}_{\text{mean}}$ = 0.66), the mean auROCs of the predicted tissue expression of pGenes across the 53 pairs (${\text{auROC}}_{\text{mean}}$ = 0.62) were significantly (Wilcoxon paired test, $P-\text{value}=4.03\times 10^{-4}$) higher than those of the original blood expression (${\text{auROC}}_{\text{mean}}$ = 0.59), suggesting that predicted tissue expression profiles were informative for disease status (Fig. 3c and d and Supplementary Fig. S15).

### 3.5 MTM facilitates the identification of tissue-specific disease-associated dysregulations

**Figure 4.**
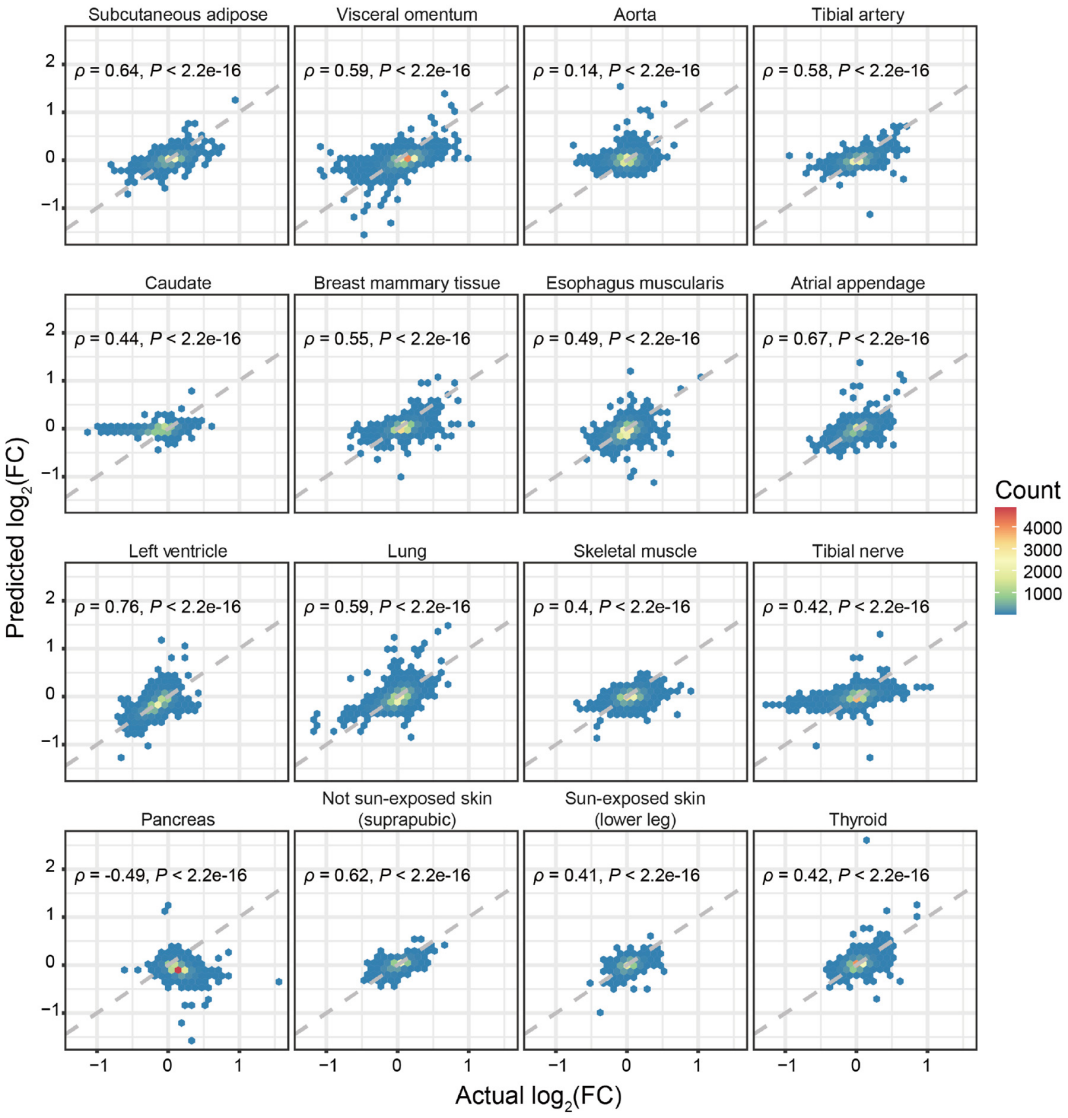
Potential utilities in analyzing disease-related gene signatures in external data. Hexagonal heatmaps show the relations between the ${\;\text{log}}_{2}$ fold changes of the predicted tissue expression using blood from external T2D patients and the reference ${\;\text{log}}_{2}$ fold changes (GTEx T2D subsets). Hexagonal cells are colored according to numbers of genes (count).

Since MTM could capture individualized biological variations from any input tissue expression profiles, we further explored the potential application scenarios for MTM. We investigated whether MTM could predict disease-related dysregulations of tissues from blood expression on an external dataset. The external blood expression data were input into MTM to predict the expression profiles of other tissues, which were then used to perform DE analysis and were compared with the GTEx reference (with at least 50 cases and 50 controls). The dataset includes 25 T2D patients and 33 normal subjects (Chen *et al.* 2022). Among 16 T2D-related tissues (with at least 10 observed up- or downregulated DEGs under FDR < 0.05 within GTEx), the predicted T2D-related expression changes (fold changes) exhibited high concordance with the reference data (Pearson’s ρ ranged from −0.49 to 0.76, median = 0.52, Fig. 4), which were significantly higher (Wilcoxon rank sum test, *P*-value = .033) than those of 11 non-T2D-related tissues (Pearson’s ρ ranged from −0.54 to 0.65, median = 0.32).

## 4 Discussion

Deciphering tissue-specific gene expression of individuals is able to provide vital clues and insights into complex traits and diseases and thus aid fundamental and clinical studies. Predicting tissue transcriptome profiles from biological information of peripheral “surrogate” samples has emerged as a promising alternative with potential utility in areas of biomedical science, especially for circumstances when tissue samples are unavailable. This work finds that, through fully leveraging information from all tissues of the same individual and the nonlinear representation capability of neural networks via the multi-task learning architecture, MTM substantially enhances performance on both overall sample levels and fine-grained gene levels. In particular, MTM identifies more predictable genes with well-preserved personalized physiological and pathological variations, providing a novel and valuable tool for tissue transcriptome-based biomedical investigations from a more comprehensive and systemic perspective. It is important to note that the medical records from GTEx are medical history records, not necessarily reflecting the disease status of the donors at the time of tissue collection, especially for acute illnesses, such as acute pneumonia. Consequently, DEGs identified using those records should be carefully considered or interpreted as DEGs related to disease history, such as adaptive changes like remodeling, fibrosis, or other long-term alterations (Garza 2015, Quinton *et al.* 2018, Jensen *et al.* 2021). However, the utilization of MTM with data from acute diseases has the potential to aid in the identification of DEGs associated with disease onset, which has potential clinical implications.

The substantial improvement in performance for MTM may be attributed to several factors. First, it is commonly recognized that an individual’s gene expression levels in different tissues are inherently linked by shared biological foundations, which could be decomposed into the static part that shares the same genetic material across tissues (i.e. GReX) and the dynamic part that is related to the temporal and spatial factors of the individual, including physiological states as well as environmental and other factors. The multi-task learning design of MTM encourages the exploitation of both the static and dynamic intrinsic relevance in all tissues from the same individual, enabling the model to extract and refine decoding rules with biological patterns and to produce better individualized representations instead of solely twisting data, thus leading to improved predictive performance, especially in tissues with smaller sample sizes. Interestingly, MTM performed worst in the two cell lines, with the lowest numbers of pGenes being captured, indicating that the integrity of internal information for the individual was lost in the cell lines that were cultured or transformed *in vitro*. Second, MTM may be more capable of capturing complex gene–gene interactions with nonlinearity and multi-task components. Last but not least, predictive information, including subtle signals, is preserved to the greatest extent in MTM, since dimension reduction is not required for transcriptomic data in large-scale neural networks driven by the backpropagation algorithm (LeCun *et al.* 2015). With these properties, the framework of MTM may serve as an effective paradigm in similar biomedical scenarios where complex intrinsic relationships underlie multiple entities, such as other large-scale omics data of different tissues or cells from the same individuals.

The high accuracy of MTM for cross-tissue expression prediction with well-preserved individualized biological variations supports its potential for biomedical applications, especially in tissue-specific biomarker discovery from readily available tissues (such as blood). However, there are still gaps that remain to be filled. A major challenge is to remove the heterogeneity in real data to align with the fixed develop sets for deployed machine learning models in practice, which imposes difficulties on existing data integration methods that cannot keep the develop sets intact. Another challenge is that the multi-task learning architecture of MTM highly depends on large-scale computational resources, which limits the incorporation of additional omics data. With the future advances of computational resources and data integration algorithms, as well as new incoming data to support continuous learning, MTM will promote real-world translational applications, such as decoding molecular mechanisms and mining clinical biomarkers.

## 5 Conclusion

In this study, we propose a deep learning-based multi-task learning framework, MTM, to predict individualized tissue gene expression profiles from any available gene expression of the same individual using a single unified model. Comparisons to the two representative approaches, MTM shows the superior performance at both the sample level and the gene level, with a larger proportion of predictable genes. Ablative study confirms that jointly leveraging cross-tissue information provides improvements beyond the modeling power of nonlinear neural networks, which might be achieved by exploiting individualized representation through our multi-task learning framework. Phenotype association analysis suggests that the predicted expression profiles preserve the individualized biological variations well, including trait-related expression changes and disease-related dysregulations. In summary, our work proves multi-task learning to be an effective strategy to utilize tissue-shared intrinsic biological relevance in the prediction of cross-tissue gene expression profiles.

## Supplementary Material

btad363_Supplementary_Data

## Data Availability

All the raw data before preprocessing are available as follows: (1) GTEx cohort with gene expression data are available in the GTEx portal (https://www.gtexportal.org/) and the dbGap (https://www.ncbi.nlm.nih.gov/gap/), and can be accessed with [dbGaP Accession phs000424.v8.p2]; (2) Data of patient and control groups for Type 2 Diabetes along with blood expression profiles are available in GEO database (https://www.ncbi.nlm.nih.gov/geo/) and can be accessed with [GSE184050].
