# Supplementary material for: MTM: a multi-task learning framework to predict individualized tissue gene expression profiles
Source: Bioinformatics. 2023 Jun 5;39(6):btad363. doi: 10.1093/bioinformatics/btad363 (PMC10278940; doi:10.1093/bioinformatics/btad363)
Supplement: btad363_Supplementary_Data [file btad363_supplementary_data.zip › Supplementary.pdf]

MTM: a multi-task learning framework to predict individualized tissue gene expression profiles - Supplementary Information

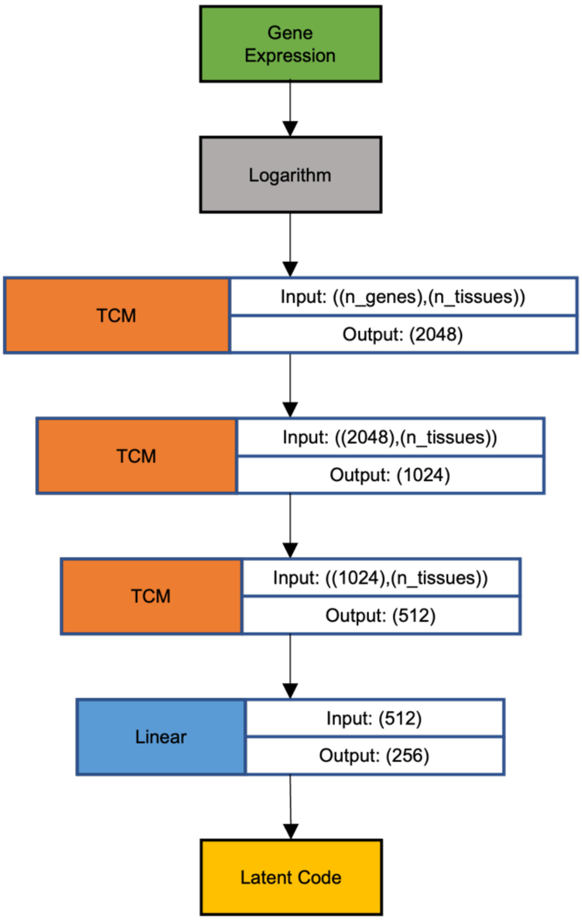

Supplementary Fig. S1. Structure of the encoder (*E*) in MTM. The encoder takes the original gene expression profiles of the source tissues and applies a logarithmic transformation. Then, tissue-conditioning modules (TCM) are used to perform tissue-specific routing and data transformation based on the tissue type. Finally, the encoder outputs a low-dimensional latent code through a linear layer.

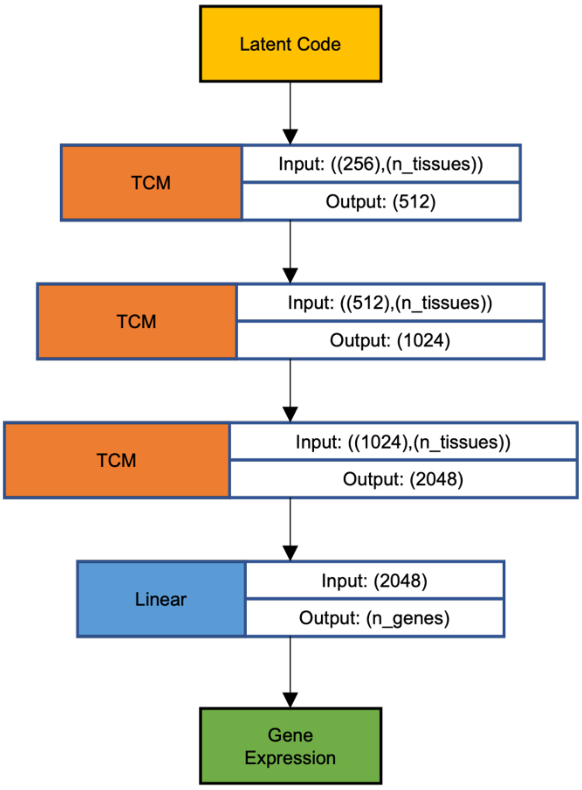

Supplementary Fig. S2. Structure of the generator (*G*) in MTM. The generator transforms the low-dimensional latent code into the gene expression profile of the target tissue through symmetric transformations that are opposite to those used by the encoder (*E*).

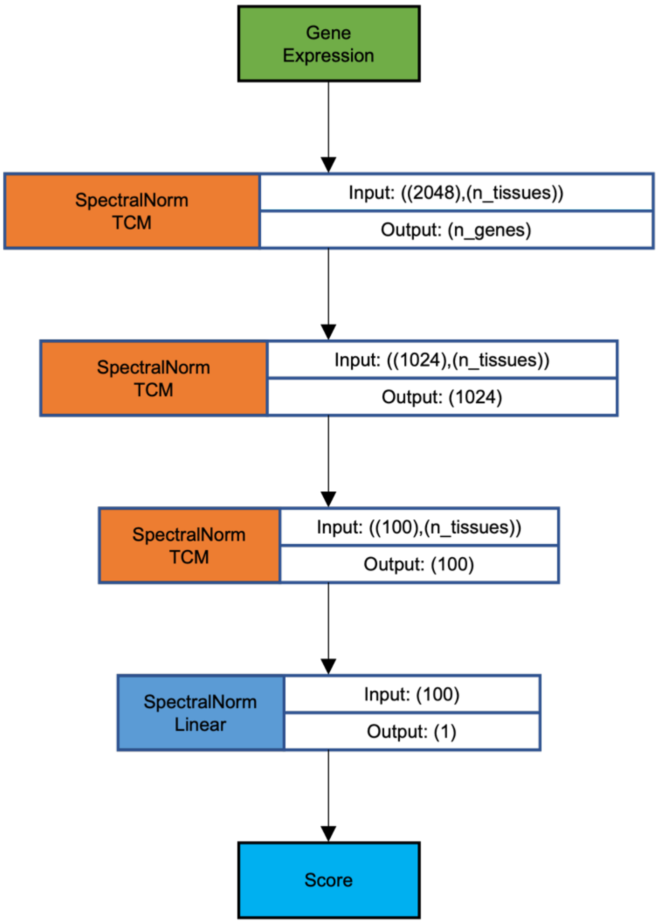

Supplementary Fig. S3. Structure of the discriminator (*D*) in MTM. The discriminator is used to determine whether the input gene expression profile of a specific tissue type is real or generated by the generator, as part of the adversarial training against the generator (*G*) to enhance the prediction performance. With the tissue-conditioning module (TCM), the discriminator is capable of scoring all types of tissues.

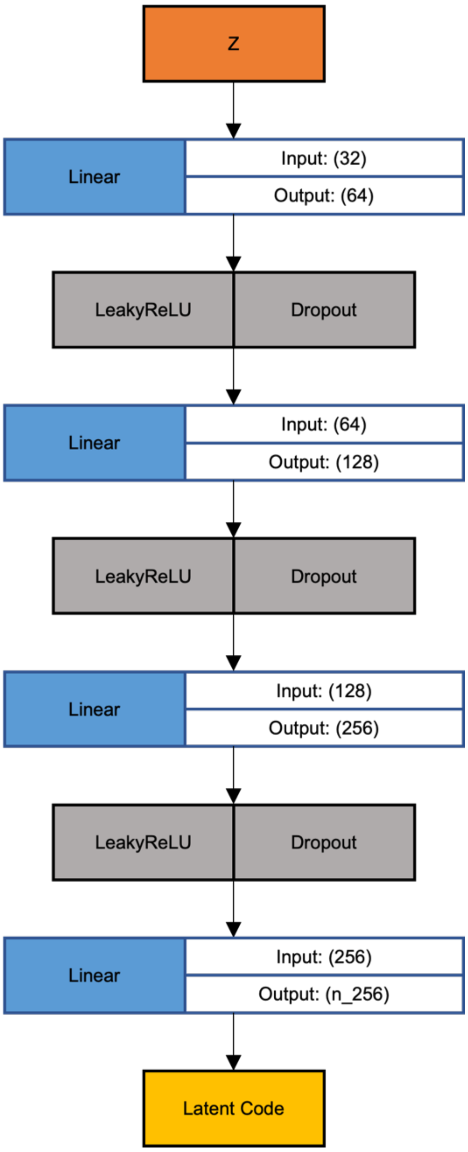

Supplementary Fig. S4. Structure of the mapping network (*M*) in MTM. The mapping network is used to convert noise *z* that follows a Gaussian distribution into a latent variable, which can be used as input to the generator (*G*) to generate a gene expression profile.

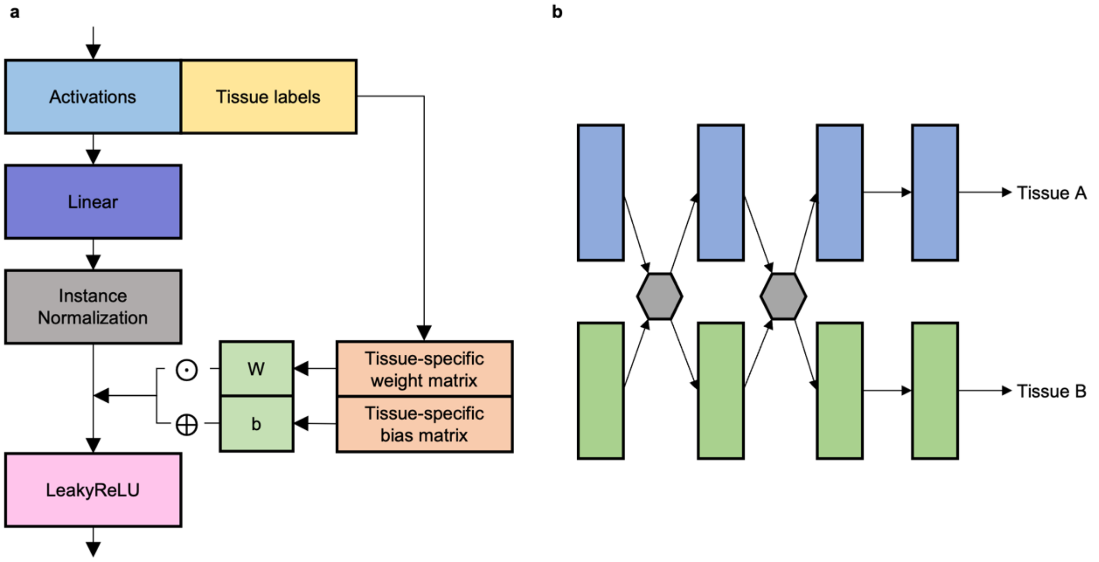

Supplementary Fig. S5. Structure of the tissue conditioning module (TCM) in MTM and illustration of parameter sharing. **(a)** TCM consists of three parts: a fully connected layer that is shared across tissue types, a learnable tissue-specific instance-level affine transformation layer, and a leaky rectified linear unit (Leaky ReLU). The purpose of the TCM is to perform routing and data transformation using tissue-specific or tissue-shared connections. **(b)** Multiple TCMs are chained together to form an alternating structure of tissue-shared and tissue-specific layers, resulting in a multi-task learning neural network based on hard parameter sharing. This design is able to capture the tissue-specific hierarchical structures, as well as the intrinsic cross-tissue biological inherence underlying gene expression profiles of multiple tissues. Grey blocks are designed to capture intrinsic inherence features that are shared across tissues, while blue and green blocks are designed to capture tissue-specific features for tissue A and tissue B.

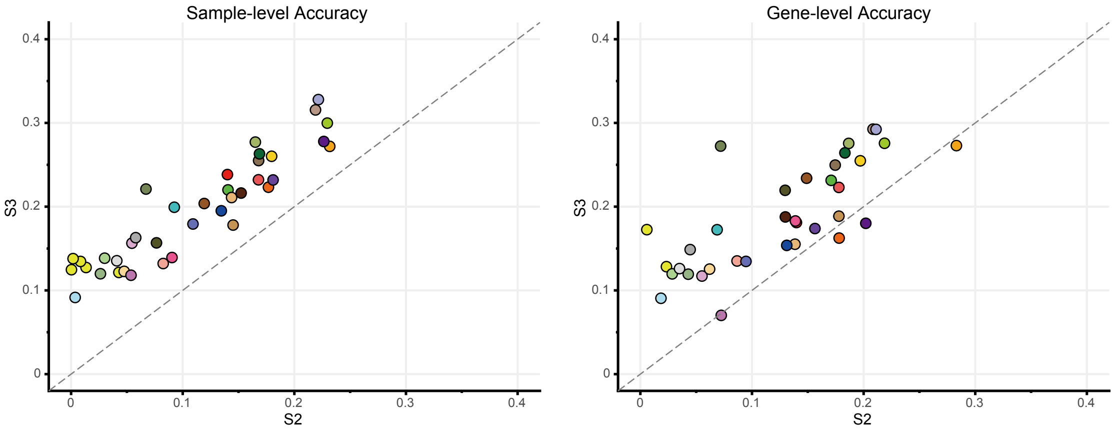

Supplementary Fig. S6. The sample-wise and gene-wise accuracy (Pearson's correlation coefficient) of S3 against S2. The values represent the average performance of all individuals (sample-level) or of all genes (gene-level) in the target tissue. Each scatter point colour represents a specific tissue as defined by the GTEx Consortium.

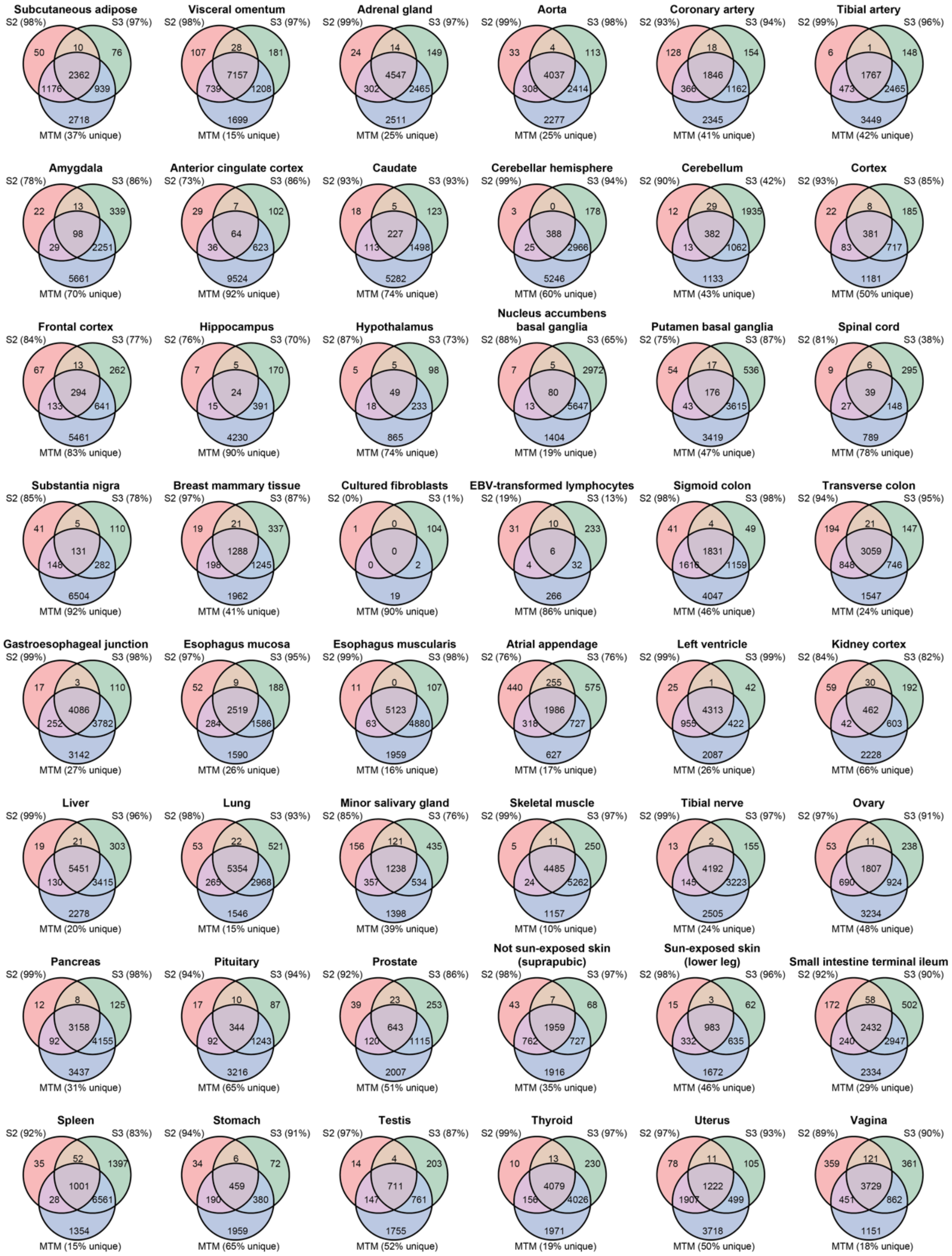

Supplementary Fig. S7. Venn diagram shows the overlap of the predictable genes (pGenes) from S2, S3 and MTM in each tissue.

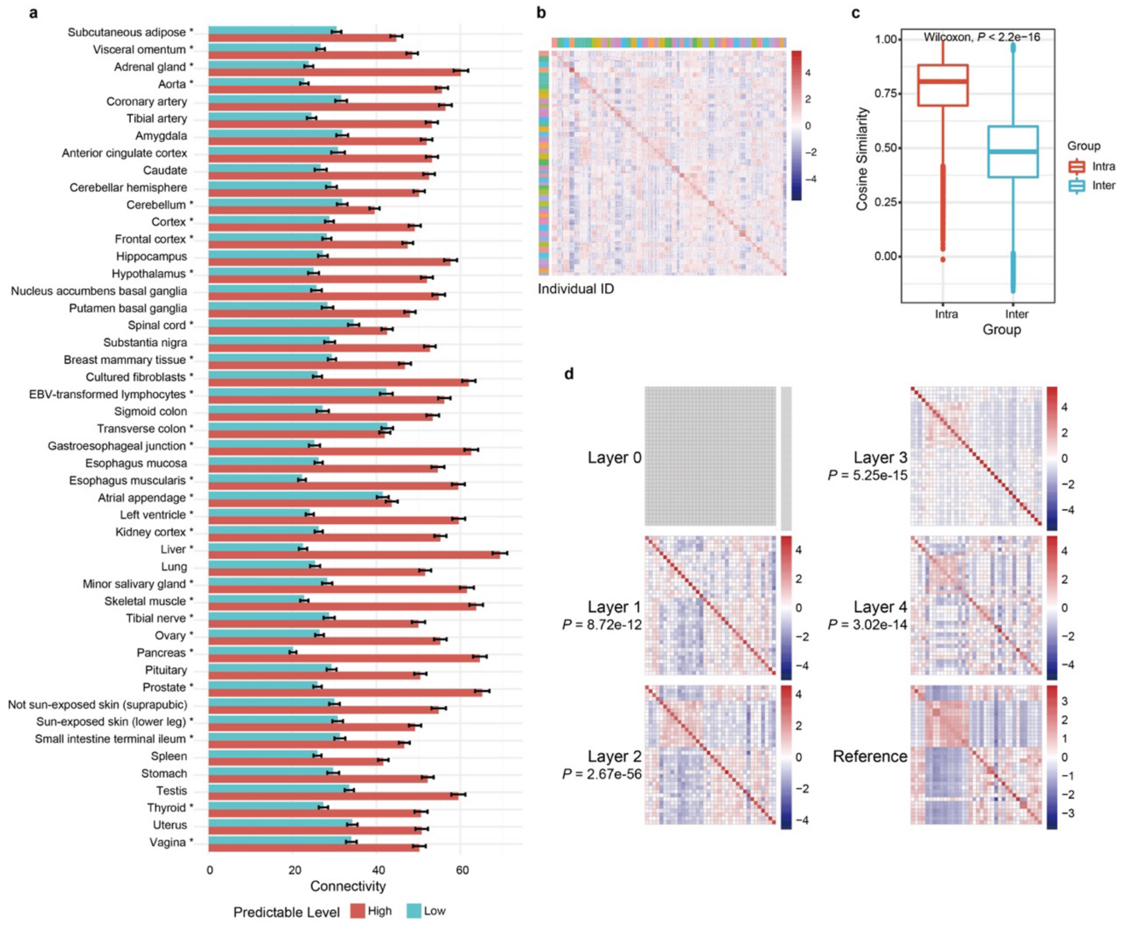

Supplementary Fig. S8. Characteristics of predicted and intermediate data derived from MTM. **(a)** Comparison of the connectivity levels (average degrees) between highly predictable (top 25 percentiles of predictable genes) and unpredictable genes (bottom 25 percentiles of predictable genes) in different tissues. Error bars represent standard error. **(b)** Heatmap displaying the pairwise similarities of latent codes of different tissues from 50 random individuals. The values of the pairwise similarities are Z-score transformed by row to reflect the scale difference in similarity levels. **(c)** Comparison of similarities of intraindividual and interindividual latent codes. **(d)** Pairwise similarities (in terms of Pearson's correlation coefficient) between different intermediate data that flow towards different target tissues in different layers of the generator (*G*) in MTM, which started from the same latent code of the blood expression from one specific individual. The values of the pairwise similarities are Z-score transformed by row to reflect the scale difference in similarity levels. Each grid is coloured to indicate a similarity level (Z-score transformed) between intermediate data of the corresponding two tissues. 'Reference' refers to pairwise similarities between the expression profiles of different target tissues. *P*-value shows the significance of Spearman's correlation analysis between vector of flattened pairwise similarities of latent codes and vector of flattened pairwise similarities of tissue gene expression profiles.

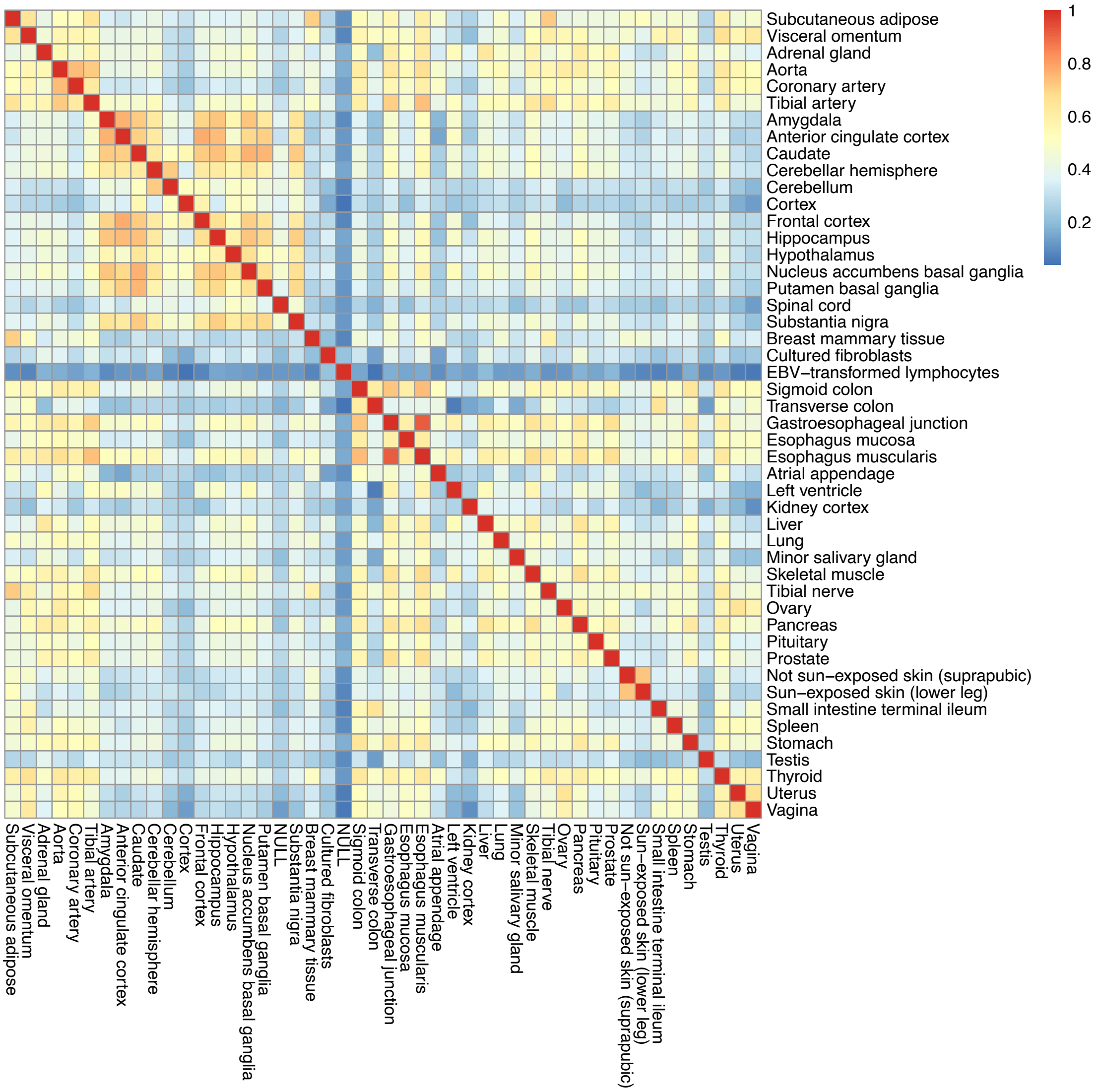

Supplementary Fig. S9. Heatmap shows the pairwise similarities of the predictability of all genes in all tissue pairs in terms of Pearson's correlation coefficients.

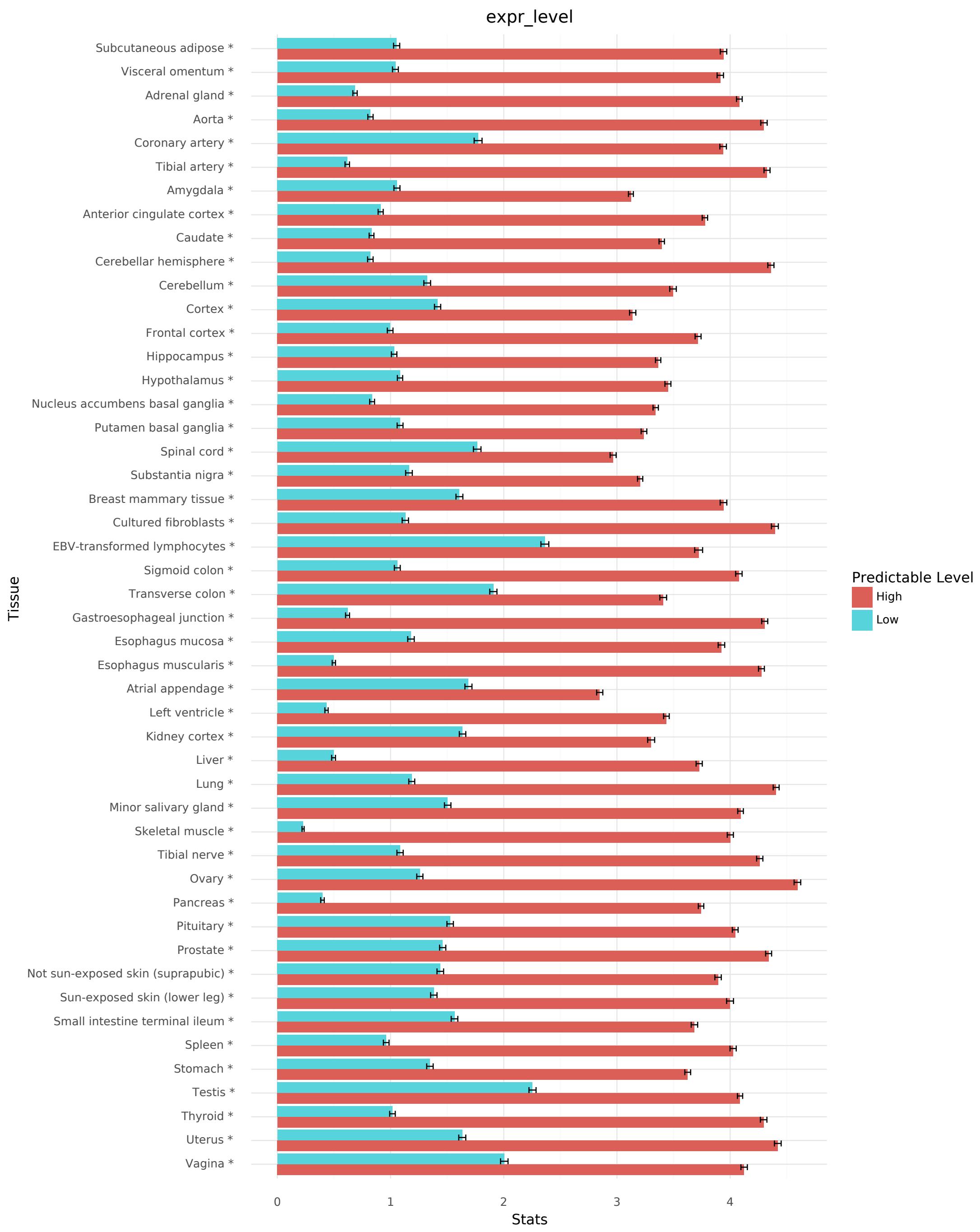

Supplementary Fig. S10. Comparison of the expression levels between highly predictable (top 25 percentile predictable genes) and unpredictable genes (bottom 25 percentile predictable genes) in different tissues. Values represent average log TPM of the highly predictable and unpredictable genes. Error bars represent standard error.

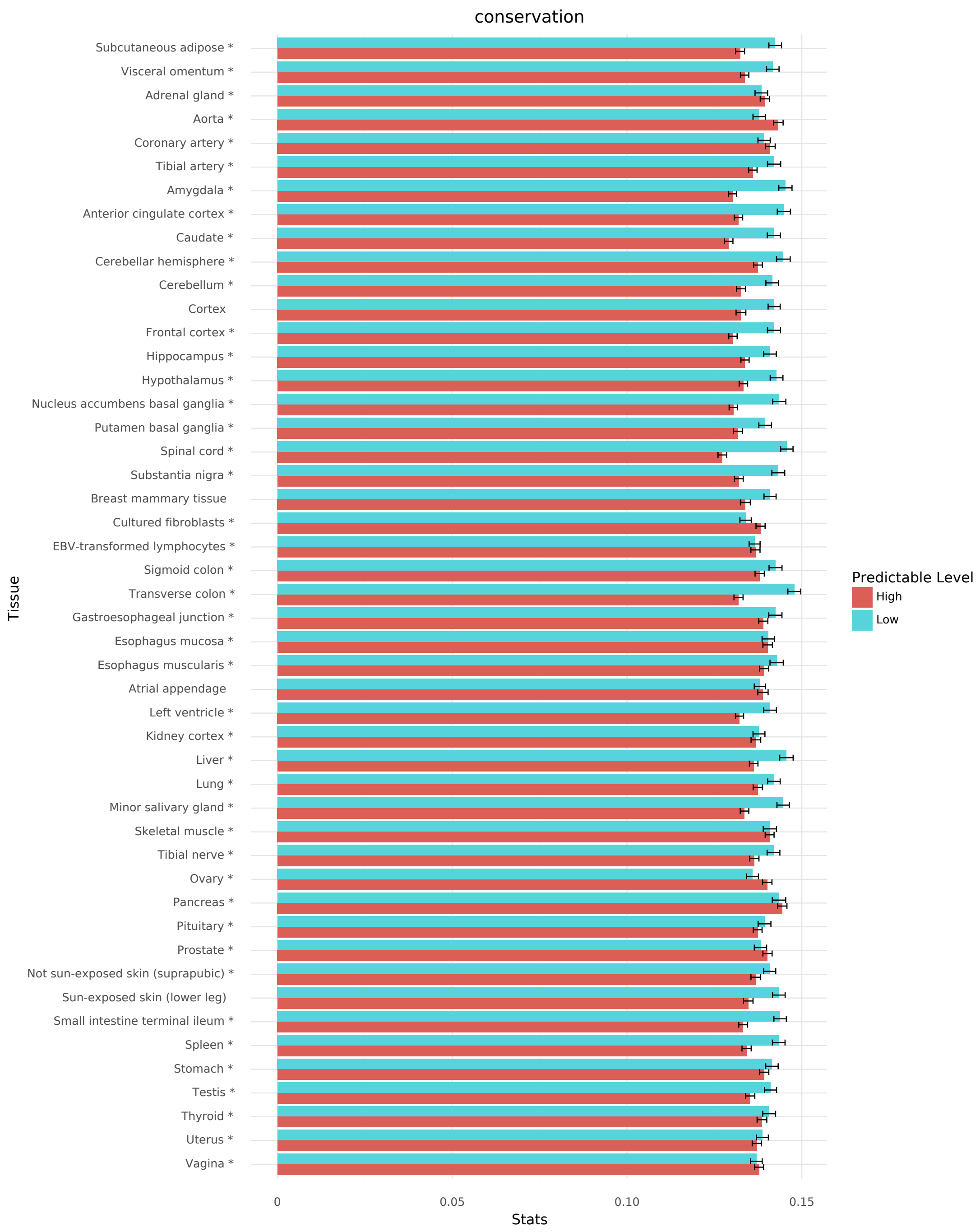

Supplementary Fig. S11. Comparison of the conservation levels (PhastCons scores) between highly predictable (top 25 percentile predictable genes) and unpredictable genes (bottom 25 percentile predictable genes) in different tissues. Error bars represent standard error.

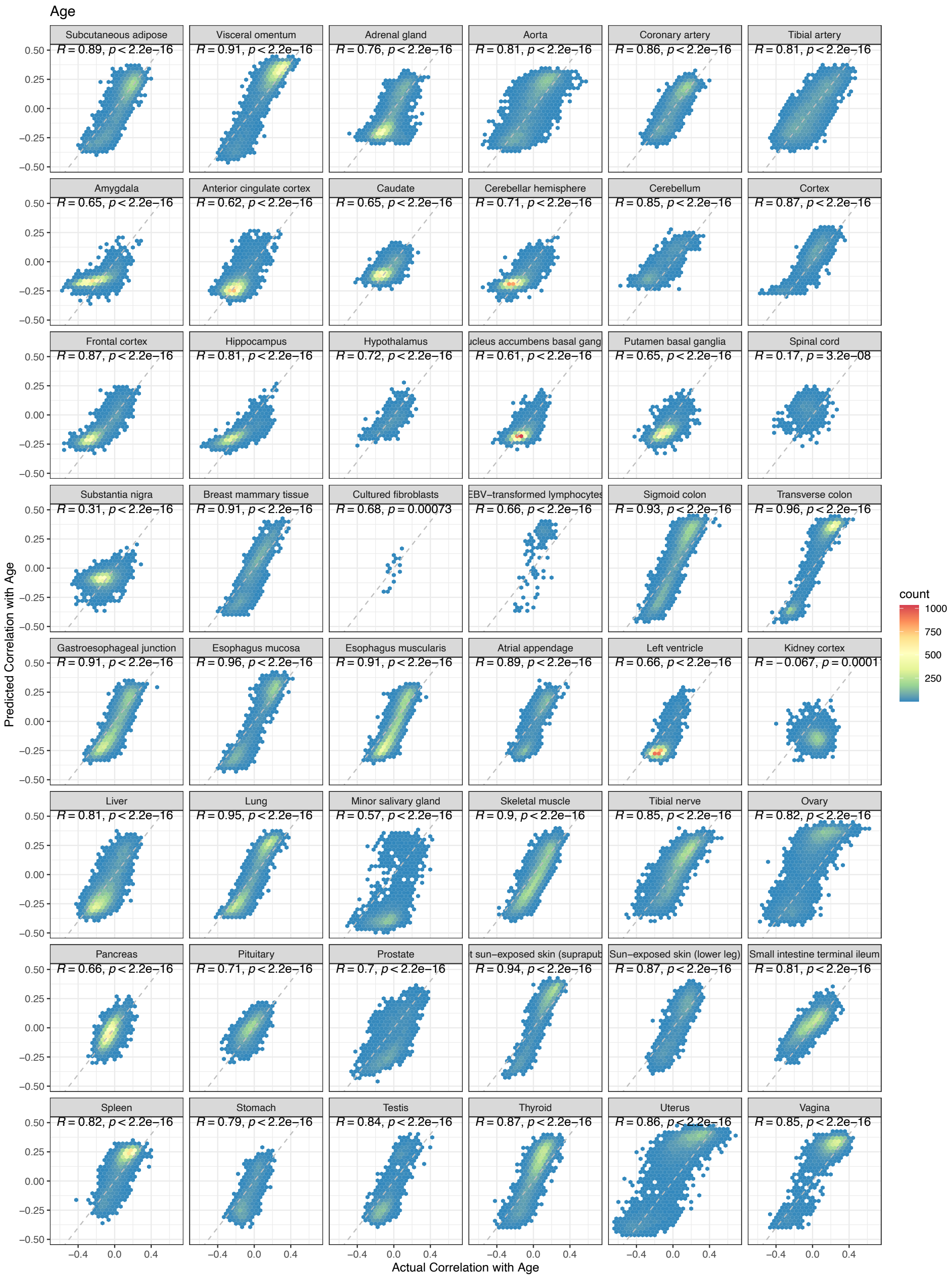

Supplementary Fig. S12. Scatter plots show relations between the associations of pGenes with age (in terms of Pearson's correlation coefficients) using predicted expression and those using the actual expression.

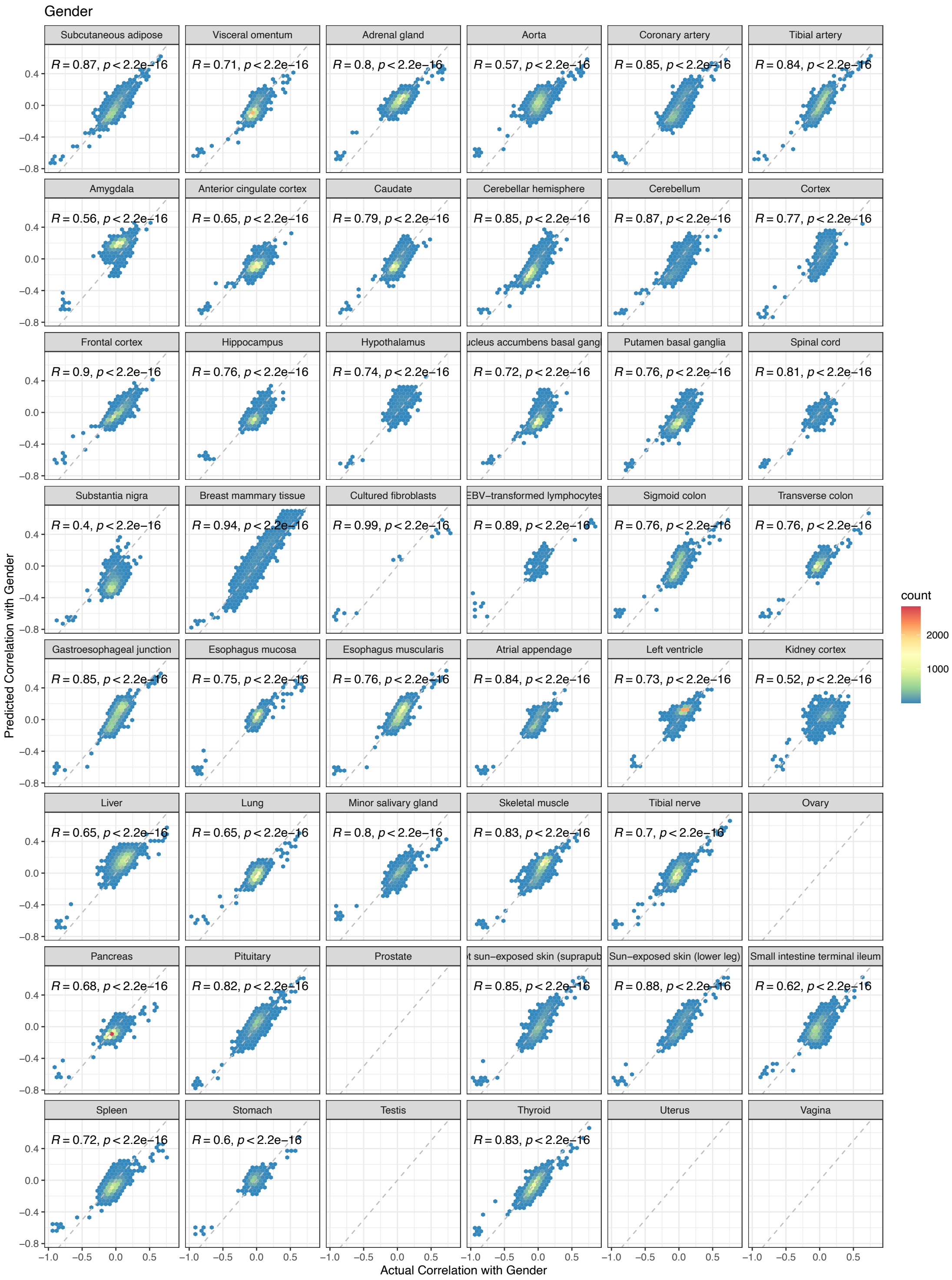

Supplementary Fig. S13. Scatter plots show relations between the associations of pGenes with gender (in terms of Pearson's correlation coefficients) using predicted expression and those using the actual expression.

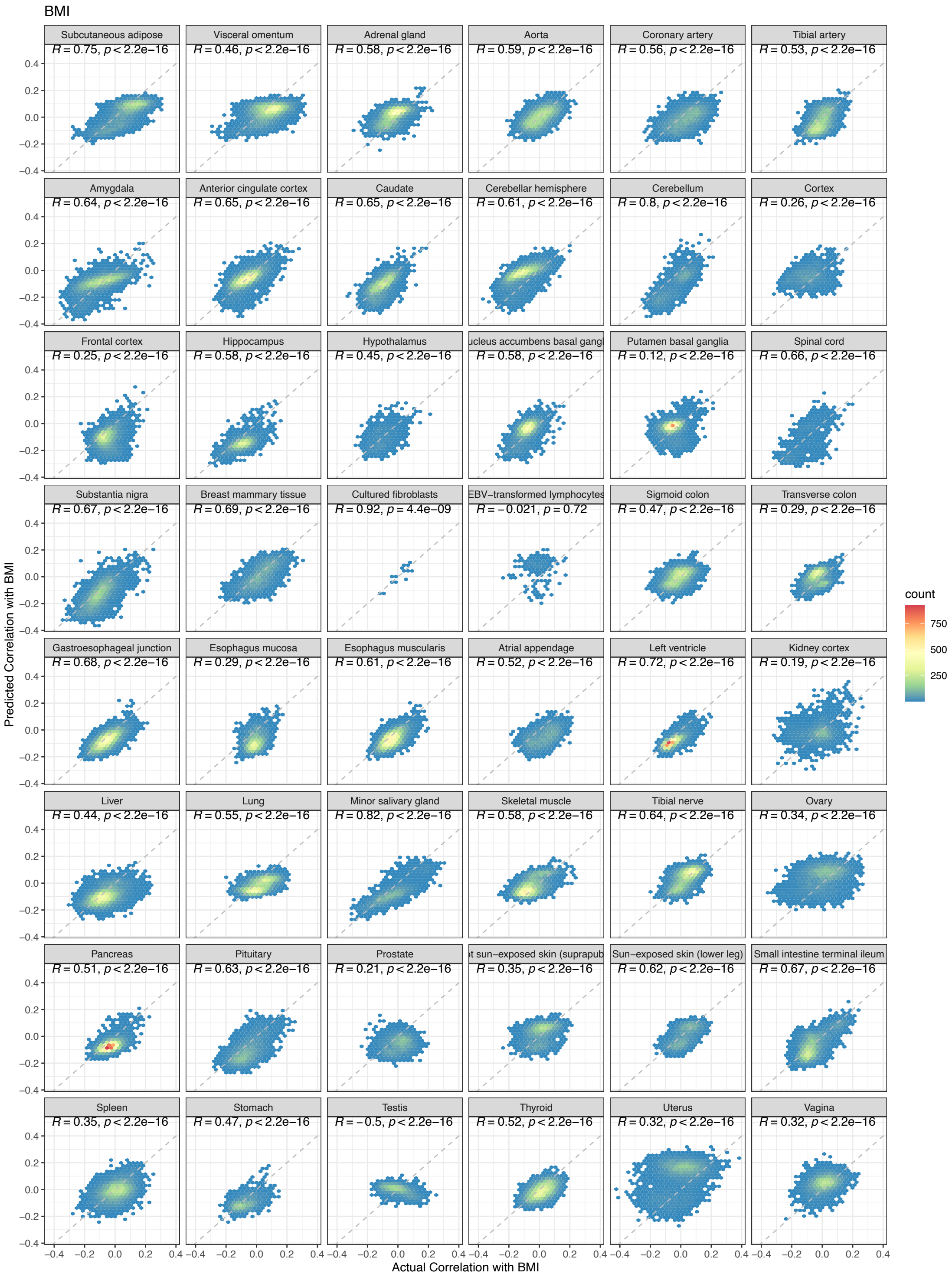

Supplementary Fig. S14. Scatter plots show relations between the associations of pGenes with BMI (in terms of Pearson's correlation coefficients) using predicted expression and those using the actual expression.

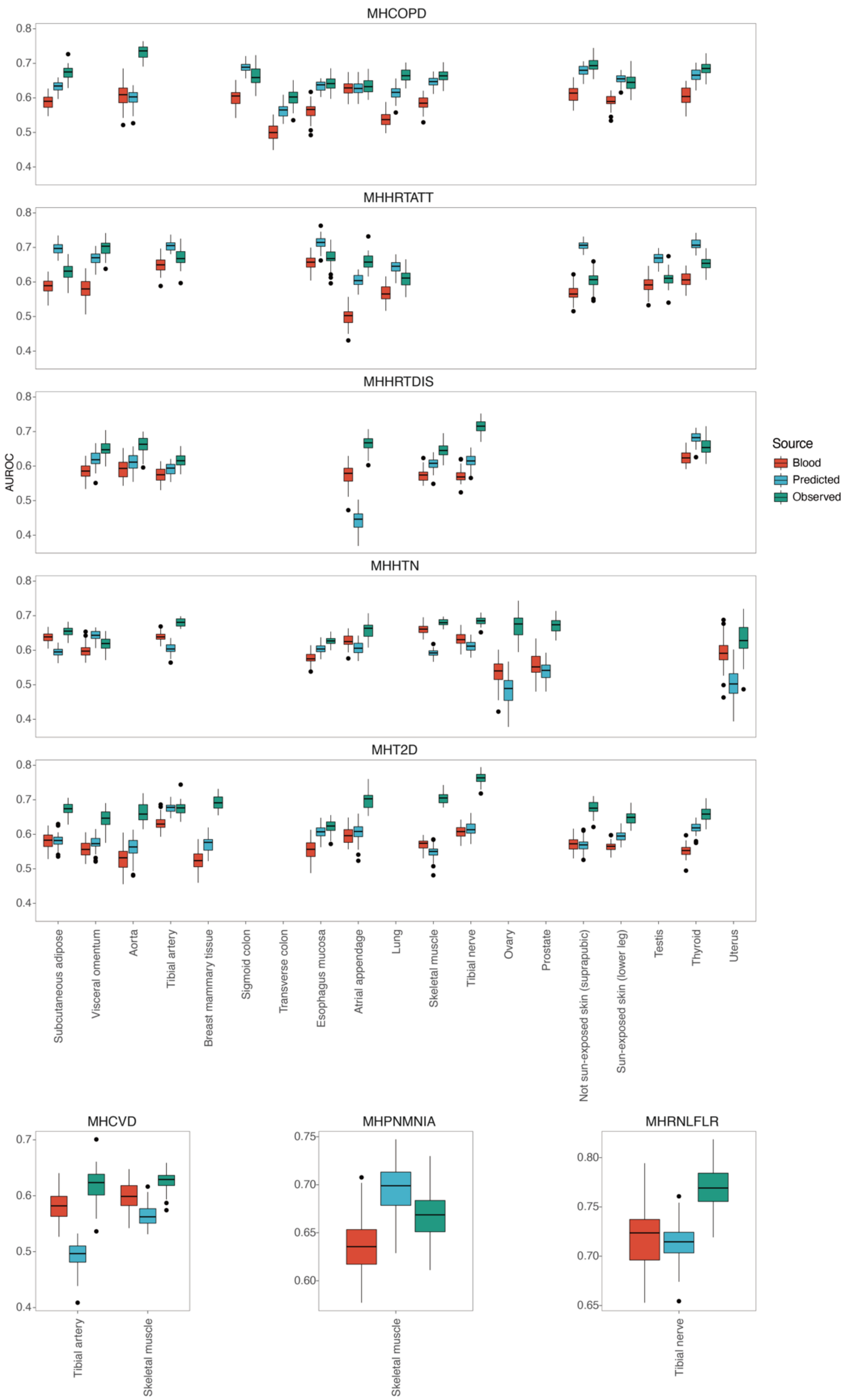

Supplementary Fig. S15. For the 53 disease-tissue pairs where the actual tissue expression profiles are more informative than those of blood, box plots show the performance of predicting disease status (in terms of auROC) using observed tissue expression, predicted tissue expression and blood expression of the pGenes in a 5-fold CV manner across 50 independent runs.
